# Changes of serum TSH, FT3, and FT4 levels in infants received surgical correction of congenital heart disease under cardiopulmonary bypass

**DOI:** 10.1186/s12872-023-03590-4

**Published:** 2023-11-16

**Authors:** Wen-Hao Lin, Si-Jia Zhou, Xiu-Hua Chen, Hua Cao, Qiang Chen

**Affiliations:** Department of Cardiac Surgery, Fujian Children’s Hospital (Fujian Branch of Shanghai Children’s Medical Center), College of Clinical Medicine for Obstetrics & Gynecology and Pediatrics, Fujian Medical University, Fuzhou, China

**Keywords:** Thyroid gland hormone, Congenital Heart Disease, Infant, Surgery, Cardiopulmonary bypass

## Abstract

**Objective:**

This study aimed to explore the fluctuations and clinical relevance of serum thyrotropin (TSH), free triiodothyronine (FT3), and free thyroxine (FT4) levels in infants undergoing surgical correction for congenital heart disease (CHD) using cardiopulmonary bypass (CPB).

**Methods:**

In a retrospective design, 58 infants who underwent CHD surgical correction under CPB between January 2021 and January 2022 at our institution were incorporated. These infants were categorized into two groups: simple CHD (n = 34) and complex CHD (n = 24). TSH, FT3, and FT4 serum concentrations were assessed at four intervals: 24 h pre-surgery (T0) and 24 h (T1), 48 h (T2), and 72 h (T3) post-surgery.

**Results:**

The simple CHD group displayed a significantly reduced CPB duration compared to the complex CHD group (P < 0.001). Both groups exhibited a notable decline in serum thyroid hormone concentrations at T1 compared to T0. However, from T1 to T3, an upward trend in hormone levels was observed. By T3, though the levels in both groups had risen notably from T1, they remained significantly diminished from T0 (P < 0.01). In both the simple and complex CHD cohorts, significant fluctuations in thyroid hormone levels (TSH, FT3, FT4) were noted across the different timepoints (T0, T1, T3) (P < 0.01). While no significant disparities were found between the two groups’ hormone concentrations at T0 and T1 (P > 0.05), at T2 and T3, the simple CHD group manifested higher TSH, FT3, and FT4 levels compared to the complex CHD group (P < 0.05).

**Conclusions:**

Infants undergoing CHD surgical correction under CPB experience significant declines in TSH, FT3, and FT4 serum levels. The post-surgery thyroid hormone recovery was more pronounced in infants with simple CHD compared to those with complex CHD. As such, vigilant monitoring of thyroid hormone levels during the perioperative phase is imperative, and timely intervention measures should be employed when necessary.

## Introduction

Congenital heart disease (CHD) is the most prevalent congenital anomaly, representing roughly 8–12% of cases globally. Surgical repair of CHD using cardiopulmonary bypass (CPB) is a cornerstone of therapy for these patients. However, evidence indicates that post-CPB, there may be disruption of the body’s innate homeostatic mechanisms. This can lead to complications like diminished cardiac output and heightened systemic vascular resistance, mirroring the features of hypothyroidism. This suggests that CPB might influence thyroid function [1]. Triiodothyronine (FT3), free thyroxine (FT4), and thyroid-stimulating hormone (TSH) play pivotal roles in cellular metabolism and hemodynamic stability. Their effects encompass increased heart rate, augmented myocardial contractility, enhanced diastolic function, decreased systemic vascular resistance, and elevated myocardial oxygen consumption [2]. Notably, FT3 has demonstrated efficacy in boosting postoperative hemodynamic function and decreasing the onset of arrhythmias during filling states. The perturbation in thyroid hormone levels induced by CPB can undoubtedly impact postoperative recovery, and in some cases, be fatal, especially in infants with vulnerable organ functionality [3, 4]. Adequate thyroid hormone levels are thus vital for post-surgical functional recovery in infants undergoing cardiac procedures. However, many infants in the immediate postoperative phase might not exhibit the classic clinical manifestations of hypothyroidism, making transient hypothyroidism easily overlooked. Hence, routine thyroid hormone monitoring is imperative for infants undergoing cardiac surgeries involving CPB. While numerous studies have elucidated the impact of CPB on postoperative thyroid hormone profiles (FT3, FT4, and TSH) in adults and older children with cardiac conditions, research focusing specifically on infants (aged < 1 year) remains limited [5, 6]. This study seeks to elucidate the alterations in serum thyroid hormone concentrations in infants post-surgical correction of CHD with CPB.

## Methods

### Patient and acography

A total of 58 infants, all under 1 year old, diagnosed with congenital heart disease (CHD) and who underwent surgical correction in our hospital between January 2021 and January 2022, were included in this study. Preoperative diagnoses were established based on the patients’ medical histories, physical examinations, and auxiliary tests. Patients were categorized into two groups, the simple CHD group, which comprised conditions such as atrial septal defect (ASD), ventricular septal defect (VSD), and pulmonary valve stenosis (PS), and the complex CHD group, including conditions like Tetralogy of Fallot (TOF), complete atrioventricular septal defect (CAVSD), total anomalous pulmonary venous connection (TAPVC), coarctation of the aorta (CoA), and interruption of the aortic arch (IAA), among others, based on their primary preoperative diagnoses and disease severity. Exclusion criteria encompassed the following: (1) Patients with concomitant organ-related diseases (such as liver, kidney, and central nervous system conditions); (2) Those with hereditary metabolic disorders; (3) Patients with pre-existing thyroid conditions or undergoing thyroid medication (including amiodarone, levothyroxine, or methimazole treatment). Gender, age, hospitalization number, body length, weight, and body mass index (BMI) data were retrieved from the hospital’s medical record system. Surgical data, including the total duration of surgery, total duration of cardiopulmonary bypass (CPB), and duration of aortic occlusion (if applicable), were recorded post-surgery. Subsequently, all infants were transferred to the Cardiac Surgery Intensive Care Unit (CICU) for specialized postoperative care, overseen by professional nursing staff. Cardiac function and ejection fraction (EF) were assessed 72 h after surgery using transthoracic echocardiography (TTE). The duration of postoperative mechanical ventilation and the administration of positive inotropic drugs (quantified using a positive inotropic drugs score) were also documented [7.

### Thyroid hormone concentrations

Blood samples were obtained from the patients at four distinct time points: 24 h before surgery (T0), 24 h after surgery (T1), 48 h after surgery (T2), and 72 h after surgery (T3). The collected blood samples were processed by centrifugation to separate the serum, which was subsequently stored at temperatures between 2 and 7 °C until further analysis. A monoband kit was utilized to determine the concentrations of FT3, FT4, and TSH in these patients. FT3 and FT4 levels were measured using a competitive enzyme immunoassay of type 5, while TSH levels were assessed through an immunoenzyme assay of type 3.

### Cardiopulmonary bypass

The cardiopulmonary bypass (CPB) procedure involved the utilization of a medical-grade PVC tube, a membrane oxygenator, and a 25 mm filter coated with heparin using the Duraflo I1 principle. A silicon pump pipe was employed for the roller pump. The CPB prefilling solution consisted of 5% albumin (10 g), 15% mannitol (2.5 mL/kg), and 10 mmol/L NaHCO_3_, with a total prefilling volume of 150 mL. St. Thomas cardioplegia and ice were employed for inducing cardiac arrest.

### Statistical analyses

Statistical analyses were conducted using SPSS 26.0 software. Measurement data were expressed as mean (x) ± standard deviation (s) and assessed for normal distribution. All measurement data were found to be in accordance with a normal distribution. The comparison between the two groups was performed using the t-test, while repeated-measure ANOVA was used for assessing differences at multiple time points within the same group. In cases where significance was observed, post hoc comparisons were made using the LSD t-test. Count data were presented as [n (%)], and comparisons between the two groups were conducted using the χ2 test. A significance level of P < 0.05 was considered statistically significant.

## Result

The patients were categorized into two groups based on their main preoperative diagnoses. The simple CHD group (n = 34) comprised 3 cases of ASD, 26 cases of VSD, and 5 cases of PS. The complex CHD group (n = 24) included 10 cases of TOF, 4 cases of CAVSD, 4 cases of TAPVC, and 6 cases of CoA/IAA. The basic patient information is presented in Table 1. All patients in this study were less than 1 year old, with the oldest being 185 days and the youngest 19 days. In the simple CHD group, the average age was 97.2 ± 47.1 days, the average body length was 57.3 ± 5.0 cm, and the average body weight was 4.8 ± 1.2 kg. In the complex CHD group, the average age was 105.5 ± 47.6 days, the average body length was 58.9 ± 5.6 cm, and the average body weight was 4.5 ± 1.5 kg. There were no statistically significant differences in these data (P > 0.05).

**Table 1:** Basic information of the research object

	The simple CHD group	The complex CHD group	P
**Gender (Boy/girl)**	24/10	15/9	0.518
**Age (days)**	97.2 ± 47.1	105.5 ± 47.6	0.513
**Height(cm)**	57.3 ± 5.0	58.9 ± 5.6	0.258
**Weight (kg)**	4.8 ± 1.2	4.5 ± 1.5	0.439
**BMI**	15.9 ± 2.0	15.1 ± 1.4	0.097
**Atrial septal defect**	3	/	/
**Ventricular septal defect**	26	/	/
**Pulmonary stenosis**	5	/	/
**Tetralogy of fallot**	/	10	/
**Complete atrioventricular septal defect**	/	4	/
**Total anomalous pulmonary venous connect**	/	4	/
**Coarctation of aorta/interruption of the aortic arch**	/	6	/

Table 2 summarizes the perioperative data. The CPB duration in the simple CHD group was 56.8 ± 8.1 min, significantly shorter than that in the complex CHD group (99.8 ± 14.9 min, P < 0.001). Moreover, the total duration of surgery, duration of ascending aorta clamp, duration of postoperative mechanical ventilation, and positive inotropic drug score in the simple CHD group were significantly lower than those in the complex CHD group (P < 0.001). Regarding the evaluation of postoperative cardiac function, the ejection fraction in the simple CHD group was significantly better than that in the complex CHD group (P = 0.003). The complex CHD group exhibited a significantly higher incidence of postoperative heart failure, pneumonia, and arrhythmia compared to the simple CHD group (P < 0.05), while there were no significant differences in the incidence of liver failure (P = 0.756) and renal failure (P = 0.374).

**Table 2:** Operation and postoperative information

	The simple CHD group	The complex CHD group	P
**Operation time (minutes)**	175.7 ± 23.3	237.7 ± 27.3	< 0.001
**CPB time (minutes)**	56.8 ± 8.1	99.8 ± 14.9	< 0.001
**Duration of ascending aorta occlusion (hours)**	23.9 ± 4.3	57.8 ± 12.6	< 0.001
**Mechanical ventilation duration (hours)**	28.6 ± 7.4	61.6 ± 10.5	< 0.001
**Inotropic score**	13.57 ± 1.88	20.51 ± 2.76	< 0.001
**Ejection fraction**			
**> 55%**	22	5	0.003
**30-55%**	9	11	
**< 30%**	3	8	
**Postoperative complications**			
**Heart failure**	3	8	0.045
**Pneumonia**	6	11	0.020
**Arrhythmia**	5	9	0.046
**Liver insufficiency**	1	2	0.756
**Renal insufficiency**	1	3	0.374

Table 3 presents the changes in serum TSH, FT3, and FT4 levels in the two groups at different time points. From T0 to T1, all three types of thyroid hormones in both groups exhibited a declining trend and increased from T1 to T3. Notably, the increase in the simple CHD group was more significant than that in the complex CHD group. There were no significant differences in serum TSH, FT3, and FT4 levels between the two groups at T0 and T1 (P > 0.05). However, at T2 and T3, the serum levels of TSH, FT3, and FT4 in the simple CHD group were higher than those in the complex CHD group (P < 0.05). One-way ANOVA along with Post Hoc analysis of the changes in TSH, FT3, and FT4 levels in the two groups at T0, T1, and T3 is presented in Table 4. The results revealed that the serum thyroid hormone levels in the patients after surgery (T1) were significantly lower than those before surgery (T0). By T3, hormone levels had significantly increased compared to those at T1 but remained significantly lower than those at T0. In both the simple CHD and complex CHD groups, the levels of thyroid hormones (TSH, FT3, FT4) varied significantly at different time points (P < 0.01).

**Table 3:** The changes of thyroid hormone levels in the subjects at different time

		The simple CHD group	The complex CHD group	t	p
**TSH**	**T0**	2.37 ± 1.30	2.42 ± 0.83	0.166	0.869
	**T1**	0.70 ± 0.29	0.80 ± 0.27	1.330	0.189
	**T2**	1.25 ± 0.13	1.12 ± 0.24	2.660	0.010
	**T3**	1.53 ± 0.19	1.36 ± 0.25	2.943	0.005
**FT3**	**T0**	4.55 ± 1.26	4.05 ± 1.08	1.577	0.121
	**T1**	1.87 ± 0.75	1.46 ± 0.55	0.954	0.344
	**T2**	2.19 ± 0.35	1.93 ± 0.56	2.175	0.034
	**T3**	3.04 ± 0.55	2.74 ± 0.52	2.092	0.041
**FT4**	**T0**	17.96 ± 3.54	16.47 ± 2.32	1.804	0.077
	**T1**	10.64 ± 1.84	10.41 ± 1.62	0.492	0.625
	**T2**	11.92 ± 1.15	11.03 ± 1.57	2.494	0.016
	**T3**	13.40 ± 1.17	12.68 ± 1.35	2.166	0.035

**Table 4:** Comparison of thyroid hormone levels at T0, T1 and T3

		Time(i)		F	p	Time(j)	Mean difference	Standard error	Significance
**Simple CHD**	**TSH**	**T0**	2.37 ± 1.30	39.287	< 0.01	**T1**	1.674	0.188	< 0.01
		**T1**	0.70 ± 0.29			**T3**	−0.829		
		**T3**	1.53 ± 0.19			**T0**	−0.845		
	**FT3**	**T0**	4.55 ± 1.26	75.077	< 0.01	**T1**	2.675	0.220	< 0.01
		**T1**	1.87 ± 0.75			**T3**	−1.172		
		**T3**	3.04 ± 0.55			**T0**	−1.503		
	**FT4**	**T0**	17.96 ± 3.54	80.637	< 0.01	**T1**	7.322	0.581	< 0.01
		**T1**	10.64 ± 1.84			**T3**	-2.812		
		**T3**	13.40 ± 1.17			**T0**	-4.510		
**Complex CHD**	**TSH**	**T0**	2.42 ± 0.83	59.128	< 0.01	**T1**	1.628	0.152	< 0.01
		**T1**	0.80 ± 0.27			**T3**	-0.560		
		**T3**	1.36 ± 0.25			**T0**	-1.068		
	**FT3**	**T0**	4.05 ± 1.08	69.425	< 0.01	**T1**	2.346	0.218	< 0.01
		**T1**	1.46 ± 0.55			**T3**	-1.041		
		**T3**	2.74 ± 0.52			**T0**	-1.305		
	**FT4**	**T0**	16.47 ± 2.32	68.661	< 0.01	**T1**	6.060	0.521	< 0.01
		**T1**	10.41 ± 1.62			**T3**	-2.279		
		**T3**	12.68 ± 1.35			**T0**	-3.781		

*The significance level of the mean difference was 0.05.

## Discussions

The body’s stress response triggered by CPB interferes with the deiodination process from T4 to T3. This results in a reduced peripheral conversion rate from T4 to T3, altering the distribution volume and shortening the half-life of T3. Consequently, this leads to a decrease in T3 synthesis without a corresponding increase in TSH levels [8–11]. FT3, the biologically active form of triiodothyronine (T3), is directly proportional to T3 levels. Its determination offers the advantage of being unaffected by changes in binding protein concentration and characteristics, making it a sensitive indicator for diagnosing thyroid function alterations. FT3 exerts a positive inotropic effect on the heart, enhancing cardiac output while reducing systemic circulation resistance [12–14]. Low postoperative FT3 levels in children with CHD can result in diminished cardiac output and hemodynamic instability during the early postoperative phase. The mechanism behind decreased FT3 levels is not entirely clear, but it may be associated with the activation of inflammatory mediators such as interleukin-6 (IL-6) and exposure to low temperatures [15]. Furthermore, factors like fasting, anesthesia, and surgical stress might contribute to the decline in FT3 levels.

CPB is a commonly employed technique in the surgical correction of CHD, yet it can disrupt the body’s normal homeostatic mechanisms, leading to various complications, including transient secondary hypothyroidism characterized by decreased circulating thyroid hormone levels. This effect is more pronounced in infants, given their relatively immature organ systems [16–18]. In our study, we observed a significant decrease in thyroid hormone levels in both groups of infants 24 h after surgery compared to preoperative level. Leebaw et al. demonstrated that transient secondary hypothyroidism could significantly impact the hypothalamus-pituitary axis, leading to reduced TSH secretion and subsequently decreased T3 and T4 levels, with these effects being exacerbated by CPB [19, 20]. In our study, all infants underwent open-heart surgery involving CPB, performed by the same CPB specialist, thereby reducing systemic procedural variations. Serum concentrations of FT3, FT4, and TSH in our study’s infants significantly decreased after cardiac surgery, beginning to rise 48 h post-surgery. By 72 h post-surgery, thyroid hormone concentrations had increased compared to levels observed at 24 and 48 h post-surgery, though they had not yet returned to preoperative levels. These findings align with the research conducted by Murzi et al., which showed trough levels of T4 and T3 in children occurring 12–48 h post-surgery and beginning to return to baseline levels 5–7 days post-surgery in the absence of complications [21]. It’s important to note that this study did not include long-term follow-up or continuous monitoring of serum thyroid hormone levels.

Thyroid hormone levels play a critical role in cardiovascular function, enhancing ischemic myocardial metabolism by regulating mitochondrial adenine nucleotide translocase activity, thereby increasing the transport of ADP into mitochondria and ATP production. Disruptions in thyroid hormone metabolism can influence surgical outcomes by impacting myocardial contraction and relaxation [22]. Acquired hypothyroidism in patients undergoing cardiac surgery can manifest as bradycardia, reduced cardiac output, contractility, and increased vascular resistance. Some infants in our study displayed symptoms of low cardiac output post-surgery, including increased heart rate, cold extremities, low blood pressure, and decreased urine output. These symptoms correlated with significant alterations in thyroid hormone levels before and after surgery. Additionally, thyroid hormones are crucial for normal neurocognitive development in infants, and even transient hypothyroidism can lead to adverse neurodevelopmental outcomes. Considering that infants with CHD are already at risk for long-term developmental delays, the detection of hypothyroidism, even if transient, has significant clinical implications [23, 24]. The observed decrease in serum thyroid hormone levels may be attributed to factors like ultrafiltration, hemodilution, and inhibition of the pituitary-thyroid axis during CPB.

Differences between our study’s results and previous research may be attributed to variations in study populations and CPB perfusion techniques. Prior studies primarily focused on adults and children with an average age above 3 years, whereas our study exclusively included infants (average age: 100.6 ± 47.0 days). Infants exhibit reduced tolerance to surgery and medications, which may explain the more pronounced hormonal changes and physiological responses observed. These findings have valuable clinical implications for guiding interventions related to thyroid hormone levels during the perioperative period in infantile patients undergoing cardiac surgery.

### Limitations

This study has certain limitations that should be acknowledged. First, the sample size in this study was relatively small, which limited the ability to compare thyroid hormone changes among different types of CHD. Additionally, thyroid hormone levels were not measured during CPB, which could provide valuable insights into the immediate effects of the procedure on hormone levels. The introduction of prefilled fluid into the CPB system might have influenced these hormone levels, and future research should consider tighter measurement intervals.

Furthermore, this study only focused on a subset of thyroid-related hormones, potentially providing a one-sided evaluation of the results. Other thyroid-related hormones that could contribute to a more comprehensive understanding of thyroid function were not included in the analysis. Moreover, the study only assessed serum thyroid hormone levels during the perioperative period and did not extend to long-term follow-up. This limits the ability to evaluate the long-term impact of thyroid hormone changes on the patients’ outcomes. Lastly, there are various factors that can influence thyroid function, and this study did not account for all potential confounding variables. Future research should aim to incorporate a broader range of factors and interventions to provide a more comprehensive understanding of the topic.

## Conclusions

Infants undergoing CHD surgical correction under CPB experience significant declines in TSH, FT3, and FT4 serum levels. The post-surgery thyroid hormone recovery was more pronounced in infants with simple CHD compared to those with complex CHD. As such, vigilant monitoring of thyroid hormone levels during the perioperative phase is imperative, and timely intervention measures should be employed when necessary.

## Data Availability

The data that support the findings of this study are available on request from the corresponding author. The data are not publicly available due to privacy or ethical restrictions.
